# Erratum: Smither, S.; et al. Effectiveness of Four Disinfectants against Ebola Virus on Different Materials. *Viruses* 2016, *8*, 185

**DOI:** 10.3390/v8080217

**Published:** 2016-08-04

**Authors:** Viruses Editorial Office

**Affiliations:** MDPI AG, Klybeckstrasse 64, CH-4057 Basel, Switzerland

The *Viruses* Editorial Office wishes to notify its readers of corrections in [1].

Page 10, the copyright statement is written as:

“© 2016 by the authors; licensee MDPI, Basel, Switzerland. This article is an open access article distributed under the terms and conditions of the Creative Commons Attribution (CC-BY) license (http://creativecommons.org/licenses/by/4.0/).”

However it should be written as:

“© Crown copyright (2016), Defence Science and Technology Laboratory (Dstl). This material is licensed under the terms of the Open Government Licence except where otherwise stated. To view this licence, visit http://www.nationalarchives.gov.uk/doc/open-government-licence/version/3 or write to the Information Policy Team, The National Archives, Kew, London TW9 4DU, or email: psi@nationalarchives.gsi.gov.uk”.

The manuscript will be updated and the original will remain available on the article webpage.
